# Exploring potential neuroimaging biomarkers for the response to non-steroidal anti-inflammatory drugs in episodic migraine

**DOI:** 10.1186/s10194-024-01812-4

**Published:** 2024-06-21

**Authors:** Heng-Le Wei, Yu-Sheng Yu, Meng-Yao Wang, Gang-Ping Zhou, Junrong Li, Hong Zhang, Zhengyang Zhou

**Affiliations:** 1 Department of Radiology, Nanjing Drum Tower Hospital Clinical College of Nanjing Medical University, Nanjing, China; 2 Department of Radiology, The Affiliated Jiangning Hospital of Nanjing Medical University, No.169, Hushan Road, Nanjing, China; 3 Department of Radiology, Nanjing Drum Tower Hospital, Nanjing, China; 4 Department of Neurology, The Affiliated Jiangning Hospital of Nanjing Medical University, Nanjing, China

**Keywords:** Migraine, Non-steroidal anti-inflammatory drugs, Multimodal magnetic resonance imaging, Machine learning, Percentage of amplitude oscillations

## Abstract

**Background:**

Non-steroidal anti-inflammatory drugs (NSAIDs) are considered first-line medications for acute migraine attacks. However, the response exhibits considerable variability among individuals. Thus, this study aimed to explore a machine learning model based on the percentage of amplitude oscillations (PerAF) and gray matter volume (GMV) to predict the response to NSAIDs in migraine treatment.

**Methods:**

Propensity score matching was adopted to match patients having migraine with response and nonresponse to NSAIDs, ensuring consistency in clinical characteristics and migraine-related features. Multimodal magnetic resonance imaging was employed to extract PerAF and GMV, followed by feature selection using the least absolute shrinkage and selection operator regression and recursive feature elimination algorithms. Multiple predictive models were constructed and the final model with the smallest predictive residuals was chosen. The model performance was evaluated using the area under the receiver operating characteristic (ROCAUC) curve, area under the precision-recall curve (PRAUC), balance accuracy (BACC), sensitivity, F1 score, positive predictive value (PPV), and negative predictive value (NPV). External validation was performed using a public database. Then, correlation analysis was performed between the neuroimaging predictors and clinical features in migraine.

**Results:**

One hundred eighteen patients with migraine (59 responders and 59 non-responders) were enrolled. Six features (PerAF of left insula and left transverse temporal gyrus; and GMV of right superior frontal gyrus, left postcentral gyrus, right postcentral gyrus, and left precuneus) were observed. The random forest model with the lowest predictive residuals was selected and model metrics (ROCAUC, PRAUC, BACC, sensitivity, F1 score, PPV, and NPV) in the training and testing groups were 0.982, 0.983, 0.927, 0.976, 0.930, 0.889, and 0.973; and 0.711, 0.648, 0.639, 0.667,0.649, 0.632, and 0.647, respectively. The model metrics of external validation were 0.631, 0.651, 0.611, 0.808, 0.656, 0.553, and 0.706. Additionally, a significant positive correlation was found between the GMV of the left precuneus and attack time in non-responders.

**Conclusions:**

Our findings suggest the potential of multimodal neuroimaging features in predicting the efficacy of NSAIDs in migraine treatment and provide novel insights into the neural mechanisms underlying migraine and its optimized treatment strategy.

**Supplementary Information:**

The online version contains supplementary material available at 10.1186/s10194-024-01812-4.

## Introduction

Migraine is the second leading cause of disability among all age groups worldwide, and the leading cause of disability among women aged 15 to 49 [1, 2]. At present, the pathophysiological mechanisms underlying migraine remain unclear. Migraine is accompanied by various clinical symptoms and can be induced by multiple factors. This suggested that migraine has complex neuropathological mechanisms, possibly leading to a far less satisfactory treatment for migraine management [3–5]. Although some studies have proposed and developed more specific treatment strategies for migraine, non-steroidal anti-inflammatory drugs (NSAIDs) are still the recommended first-line medications for the acute phase of migraine attacks [6–9]. At present, the selection strategy for NSAIDs is mainly based on the trial-and-error method, which may prolong the disease course and increase the cost of ineffectiveness. Moreover, the use of NSAIDs increases the risk of developing gastrointestinal symptoms, liver and kidney impairment, and cardiovascular accidents [10, 11]. Considering the socioeconomic influence and personal burden caused by migraine, exploring effective treatment strategies for this condition is a core area of clinical issues.

Currently, the trigeminovascular system (TVS) is widely recognized as the primary neuropathological mechanism underlying migraine [12, 13]. Multimodal magnetic resonance imaging (MRI) provides a novel approach to comprehensively gain a deeper understanding of the neuropathological mechanism and clinical treatment strategies for migraine. Disturbances in brain function and structure within the TVS contribute to the occurrence and development of migraine, and give rise to the manifestation of complicated clinical symptoms associated with migraine [14, 15]. In particular, the brain stem, thalamus, limbic system, and higher sensory cortex are the key cerebral regions involved in the TVS [13, 16]. Of note, the periaqueductal gray within the brainstem is believed to be a trigger area for migraine. This finding is strongly supported by a positron emission tomography study in which the authors observed increased cerebral blood flow in the dorsal pons during spontaneous migraine attacks [17]. On the other hand, the thalamus serves as the tertiary structure of the TVS, receiving pain signals transmitted from the dura and spinal cord trigeminocervical complex before projecting them to the high-level cortex [13]. Furthermore, migraine has been proposed as a disorder of the neurolimbic pain network [18], in which neural activity reflects the affective experience that activates our response to pain [19]. The inhibition of neural activity and a decrease in cerebral blood flow in the higher cerebral regions of the TVS may play an essential role in the neural mechanism underlying medication-induced analgesia [20, 21]. Despite these findings, the relationship between the functional and structural changes in the brain regions of the TVS and clinical treatment outcomes for migraine remains unclear.

Individualized treatment is an important trend in future medical therapy [22, 23]. The widespread use of medical imaging information for diagnosing neurological and psychiatric disorders has facilitated the development of biomarkers for migraine based on its neuropathophysiological mechanisms. These predictors can aid in disease diagnosis and classification, and help predict the outcomes and prognosis of individualized treatment [15, 24–28]. Neural activity in the cerebral cortex can be used to effectively classify migraine and its subtypes [24, 25]. Furthermore, prediction models for assessing acupuncture efficacy have been established based on functional and structural changes in patients with migraine, which have yielded promising results [26, 27]. However, few studies have been conducted using multimodal MRI information to predict the efficacy of NSAIDs in migraine management.

Machine learning (ML), a branch of artificial intelligence, has emerged as a powerful tool for analyzing complex data sets and making predictions based on identified patterns. By integrating multiple neuroimaging features and leveraging the power of ML algorithms, it is possible to construct models that can predict treatment outcomes with high accuracy. In this study, we hypothesized that abnormalities in the percent amplitude of fluctuation (PerAF) and gray matter volume (GMV) hold predictive value for the efficacy of NSAIDs in patients with migraine. PerAF is indicative of the magnitude of spontaneous neuronal activity and is not influenced by the scale of the original signal, showing higher reliability [29]. Concurrently, GMV offers insights into the structural integrity of brain regions. Such a model could guide clinicians in making informed decisions on NSAIDs prescriptions, aiming to optimize treatment outcomes and reduce reliance on trial-and-error methods.

## Methods

### Participants

Patients diagnosed with episodic migraine conforming to the International Classification of Headache Disorders (the 3rd Edition) [30] were recruited between January 2018 and December 2023. The diagnosis was made by a qualified neurologist based on a detailed medical history, clinical examination, and exclusion of other potential causes of headache. Inclusion and exclusion criteria for patients with episodic migraine and MRI scanning parameters are provided in detail in Supplementary Materials. Ethical approval was obtained through the Ethics Committee of the Affiliated Jiangning Hospital of Nanjing Medical University (20180285, 2020–03-026-K01, and 2023–03-010-K01). Informed consent was obtained from all subjects according to institutional review board–approved protocols, which were carried out in accordance with the Declaration of Helsinki.

Patients with episodic migraine have been required to complete comprehensive structured questionnaires before the scanning, including demographic data (e.g., age, sex, and education level), migraine-related features (e.g., disease duration, frequency, attack time, headache intensity, impact extent, and burden on quality of life), neuropsychiatric assessment (e.g., anxiety, depression, and sleep disorders), and sleep quality. Specifically, the intensity, extent, and burden of migraine were assessed using the Visual Analogue Scale (VAS) [31], Headache Impact Test 6-Item (HIT-6) [32], and Migraine Disability Assessment Scale (MIDAS) [33], respectively. Anxiety, depression, and sleep quality were measured using the Generalized Anxiety Disorder 7-Item (GAD-7) [34], Patient Health Questionnaire 9-Item (PHQ-9) [35], and Pittsburgh Sleep Quality Index (PSQI) [36], respectively.

## Response assessment

After scanning, patients with episodic migraine were asked to record the headache diary, mainly including the date of migraine attacks, the types and doses of medications taken, and the intensity of pain experienced before and 2 h after medication administration over the following 3 months. The documentation of date allows us to quantify the frequency and number of attacks, while the recorded levels of pain serve as a critical indicator for evaluating the treatment efficacy. The criteria for evaluating efficacy were as follows: (1) no pain after 2 h; (2) improvement from moderate to severe pain to mild or no pain (or decrease in VAS score by 50%) after 2 h; (3) no recurrence or need for medications within 24 h after successful treatment; and (4) repeatable curative effects with effects in at least more than two of the three attacks.

## Data processing

The processing of functional images was performed using the Resting-state Functional MRI Data Analysis Toolkit plus (RESTplus, http://restfmri.net/forum/) [37]. The main steps were as follows: (1) The first 10 time points were discarded to avoid instability of the initial MRI signals, (2) slice-timing adjustment and realignment, (3) spatial standardization into Montreal Neurological Institute (MNI) space (resampling voxel size = 3 mm × 3 mm × 3 mm), (4) smoothing with a full-width at half-maximum (FWHM) of 6 mm and detrending, and (5) controlling head motion, white matter (WM) signal, and cerebrospinal fluid signal (CFS). Participants who exhibited head motion of less than 2.0 mm displacement or a 2.0◦ rotation in any direction were included. For PerAF calculation, bandpass filtering was performed with a frequency range of 0.01–0.08 Hz. The detailed formula calculating the PerAF value of a single voxel is referred to Jia et al. [29].

The processing of 3D T1-weighted images was performed using the Voxel-Based Morphometry toolbox (VBM8, http://dbm.neuro.uni-jena.de/vbm) based on the Statistical Parametric Mapping (SPM12, https://www.fil.ion.ucl.ac.uk/spm/). The structural images were first visually screened to exclude poor-quality images and then segmented into WM, GM, and CSF using the standard segmentation model. After affine registration of the GM concentration map to MNI space, the images were nonlinearly deformed using the DARTEL algorithm and resampled to 1.5 mm^3^ isotropic voxel size. The normalized and modulated tissue probability map of GM volume was obtained by multiplying the GM concentration map by the nonlinear determinant. Finally, the GM images were smoothed with a FWHM of 15 mm.

## Model construction

The propensity score matching (PSM) was employed to account for any discrepancies in the baseline data between patients with response and nonresponse to NSAIDs in terms of demographic data, migraine-related features, and neuropsychiatric assessment. The final included patients with episodic migraine after PSM analysis were allocated to training and testing cohorts in a ratio of 7:3, using a stratified random sampling method. Further, the least absolute shrinkage and selection operator (LASSO) logistic regression and support vector machine recursive feature elimination (SVM-RFE) algorithms were used for feature selection.

The included neuroimaging features were used for constructing multiple ML models including logistic regression, SVM, random forest (RF), decision tree (DT), k-nearest neighbor (KNN), multilayer perceptron, elastic network, light gradient boosting machine (Lightgbm), extreme gradient boosting algorithms. The tenfold CV on the training data was conducted to prevent overfitting, followed by a calibration curve. The Brier score was applied as the calibration index wherein lower Brier scores represent a higher calibration degree. The model with the lowest root mean square (RMS) of residuals and Brier score was selected as the final model.

Seven statistical metrics were utilized to evaluate the model performance: area under the receiver operating characteristic (ROCAUC) curve, area under the precision-recall curve (PRAUC), balance accuracy (BACC), sensitivity, F1 score, positive predictive value (PPV), and negative predictive value (NPV).

Moreover, Shapley additive explanations (ie, SHAP values) [38] were calculated to further quantify the association of a variable with the outcome individually. A positive SHAP value indicates that the corresponding variable tends to response, and vice versa. The contribution of a variable toward prediction performance is represented by the magnitude of SHAP values.

## External validation cohort

For external validation, we used a public database: the OpenNEURO database (https://openneuro.org/datasets/ds000208/versions/1.0.1) [39]. The external validation cohort consists of 39 patients with chronic osteoarthritis pain, with an average age of 58.36 years and a standard deviation of 7.47. The gender distribution is nearly balanced with 18 males and 21 females. Patients with chronic osteoarthritis pain were treated with either placebo or duloxetine. Ultimately, 18 patients experienced a beneficial response to treatment, while 21 did not. Among the 20 patients who received duloxetine, 8 patients reported beneficial response.

## Subgroup analysis and correlation analysis

All continuous variables were grouped according to the medians. The interaction effects were assessed via subgroup analysis. Additionally, we calculated the contribution levels of features in age and sex subgroups to evaluate the potential bias of age and sex that could act as confounding factors in the association between features and outcomes.

Then, Spearman correlation analysis was performed to analyze the correlation between the included neuroimaging features and clinical characteristics of patients with episodic migraine.

## Statistical analysis

**Fig. 1:**
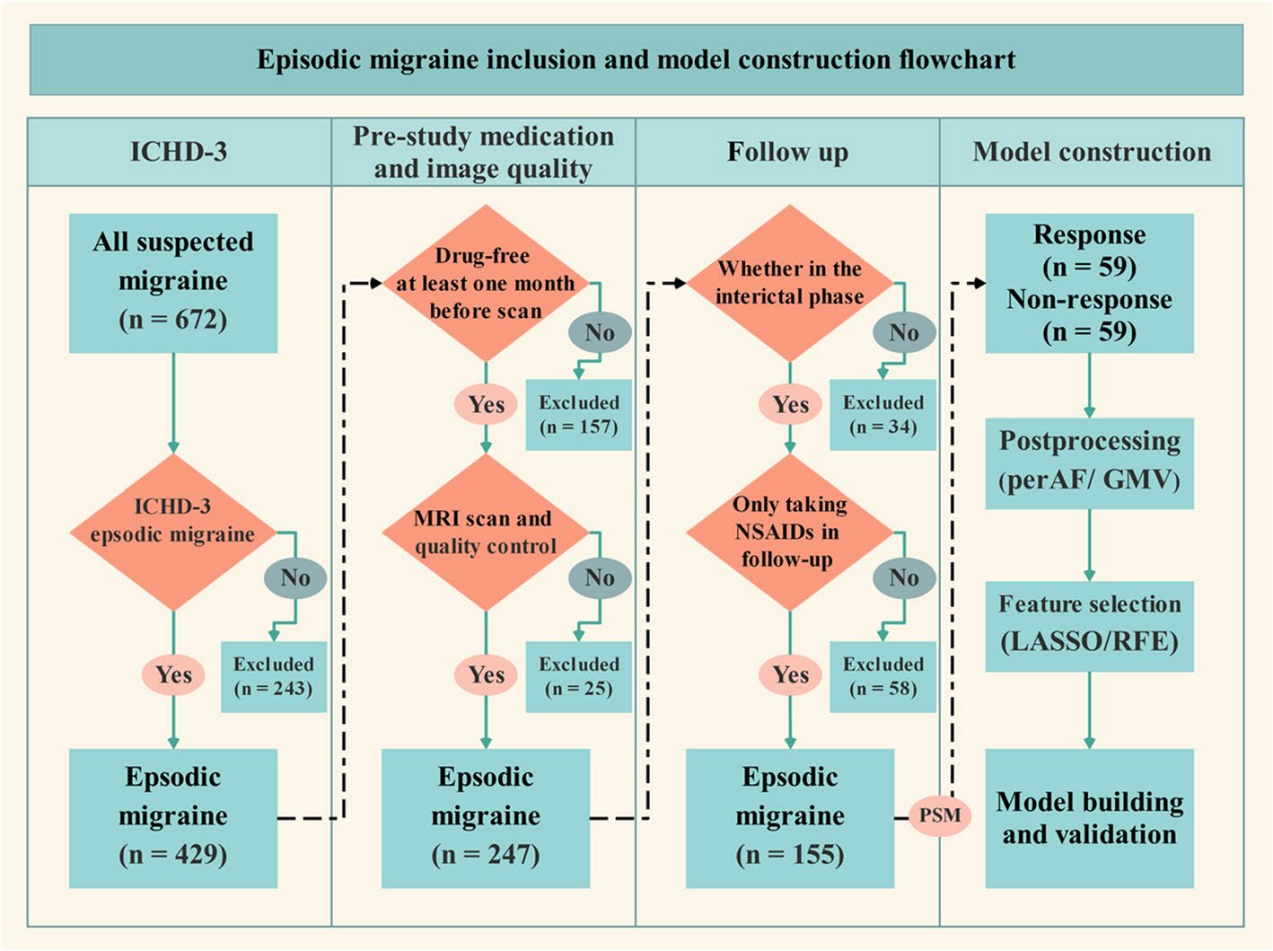
The flowchart illustrates all processes of patient selection and model construction. GMV, gray matter volume; ICHD, International Classification of Headache Disorders; LASSO, least absolute shrinkage and selection operator; NSAIDs, non-steroidal anti-inflammatory drugs; PerAF, percent amplitude of fluctuation; PSM, propensity score matching; RFE, recursive feature elimination

Statistical analyses were performed using SPSS software (version 24.0) and R software (version 4.2.1). Continuous variables with normal distribution were presented as mean (standard deviation), whereas those with non-normal distribution were presented as median (interquartile range) and compared using Mann–Whitney U test. Categorical variables were analyzed using Chi-square or Fisher’s exact tests. Statistical significance was established at a threshold of *p* < 0.05. The flowchart presented in Fig. 1 describes all processes of sample selection and model construction.

## Results

### Demographic and clinical characteristics

After PSM, 118 patients with migraine (59 responders and 59 non-responders) were included in this study. No significant differences were found in the demographic data, migraine-related features, and neuropsychiatric assessment between the patients with response and nonresponse to NSAIDs (Table 1).

**Table 1:** Comparisons between patients with response and non-response to NSAIDs after PSM

	Responders (*n* = 59)	Non-responders (*n* = 59)	*z/χ*^*2*^	*p* value
Age (years)	35.00 (16.50)	33.00 (15.50)	-0.059	0.953
Sex (female/male)	49/10	49/10	0.000	1.000
Education (years)	12.00 (7.00)	12.00 (4.00)	-0.073	0.942
Disease duration (years)	8.00 (14.00)	7.00 (12.00)	-0.197	0.844
VAS (scores)	6.00 (2.00)	6.00 (2.50)	-0.120	0.904
Frequency (days/month)	3.00 (2.50)	4.00 (2.00)	-0.027	0.978
Attack time (hours)	15.00 (12.00)	12.00 (16.00)	-1.255	0.209
MIDAS (scores)	20.00 (35.00)	21.00 (35.00)	-0.226	0.821
HIT-6 (scores)	62.00 (15.00)	61.00 (15.00)	-0.299	0.765
GAD-7 (scores)	5.00 (6.00)	6.00 (3.00)	-0.124	0.901
PHQ-9 (scores)	7.00 (8.00)	7.00 (3.50)	-0.443	0.658
PSQI (scores)	7.00 (6.50)	7.00 (4.50)	-0.311	0.756

Continuous variables conforming to the normal distribution are presented as mean (standard deviation); and continuous variables conforming to the non-normal distribution are presented as median (interquartile range)

*GAD-7* Generalized Anxiety Disorder 7-Item, *HIT-6* Headache Impact Test 6-Item, *MIDAS* Migraine Disability Assessment Scale, *NSAIDs* non-steroidal anti-inflammatory drugs, *PHQ-9* Patient Health Questionnaire 9-Item, *PSM* propensity score matching, *PSQI* Pittsburgh Sleep Quality Index, *VAS* Visual Analogue Scale

### Model performance

**Fig. 2:**
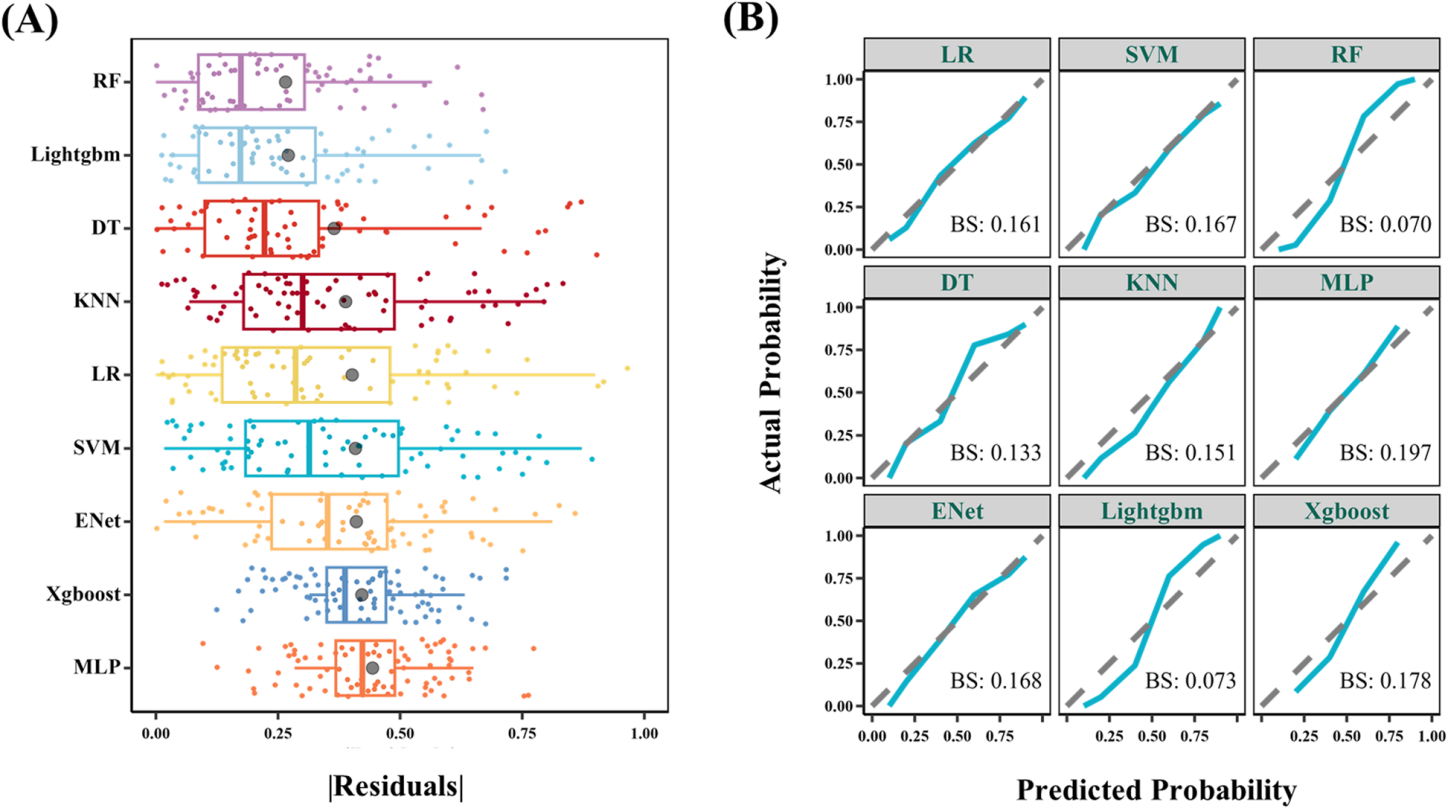
Residual plots for all models to demonstrate the residual distribution. Grey dots represent root mean squares (**A**). Calibration curves and Brier scores of all models (**B**). BS, Brier score; DT, decision tree; ENet, elastic network; RF, random forest; KNN, k-nearest neighbor; Lightgbm, light gradient boosting machine; LR, logistic regression; MLP, multilayer perceptron; SVM, support vector machine; Xgboost, extreme gradient boosting

**Fig. 3:**
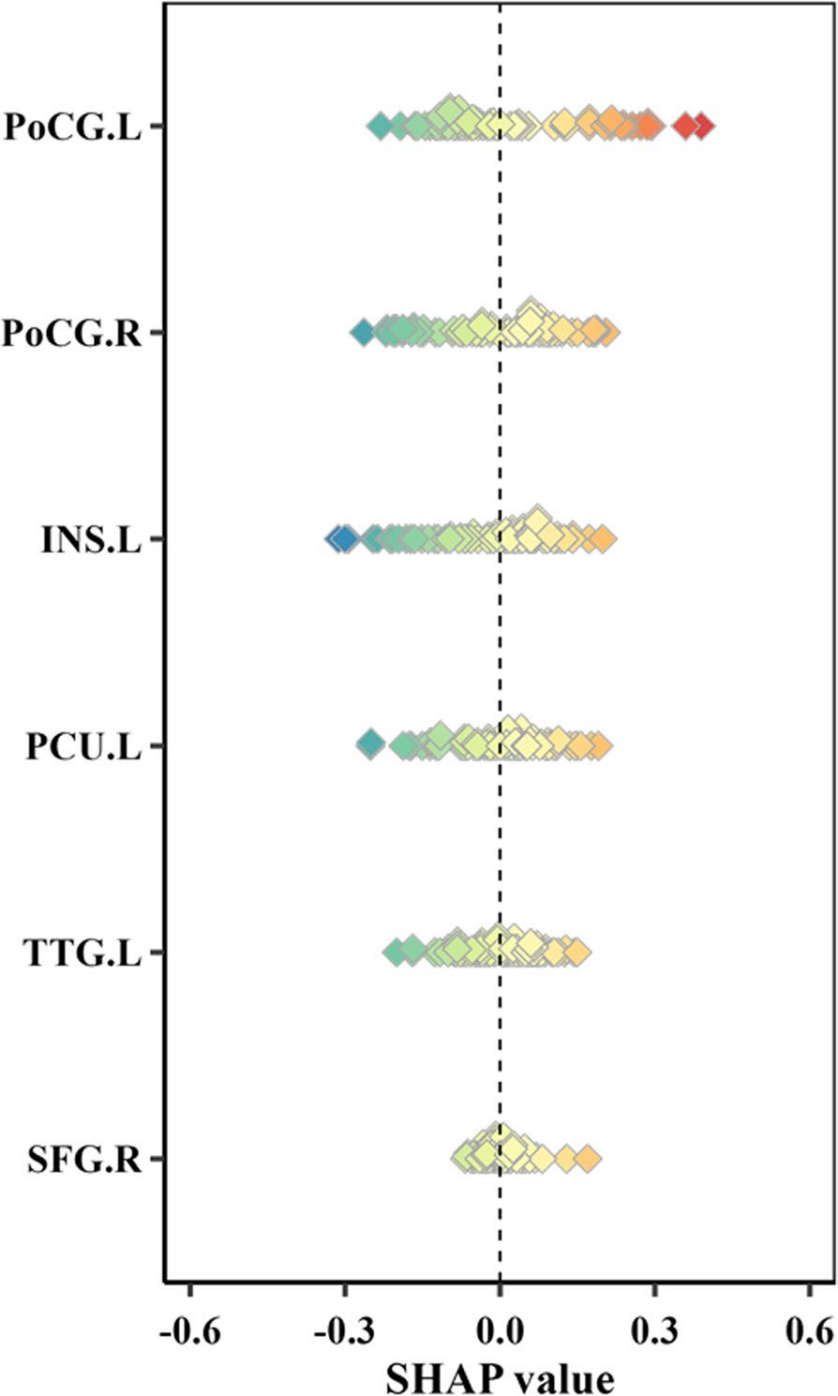
SHAP values illustrate how features contribute to the random forest model. Blue shows a negative contribution, and orange shows a positive contribution. The mean of SHAP values indicates the average contribution of the variable in the model and is provided on the vertical axis in descending order. INS, insula; PCU, precuneus; PoCG, postcentral gyrus; SHAP, SHapley Additive exPlanations; SFG, superior frontal gyrus; TTG, transverse temporal gyrus; L, left; R, right

The patients with episodic migraine were randomly divided into a training group (*n* = 82) and a testing group (*n* = 36) according to a ratio of 7:3. Six features were finally included after LASSO and SVM-RFE algorithms, namely the PerAF of the left insula (INS.L) and left transverse temporal gyrus (TTG.L), and the GMV of the left postcentral gyrus (PoCG.L), right PoCG (PoCG.R), left precuneus (PCU.L), and right superior frontal gyrus (SFG.R). The RF model was selected as the best model based on its lowest RMS (Fig. 2A) and Brier score (Fig. 2B). Figure 3 displays the contribution importance of the selected features in RF model to the identification ability on the vertical axis in descending order (PoCG, INS, PCU, TTG, and SFG) and the distribution of SHAP values of a single feature in an individual. In particular, the GMV of PoCG.L plays a significantly positive role in treatment response, whereas the PerAF of INS.L exhibits an inverse effect.

**Fig. 4:**
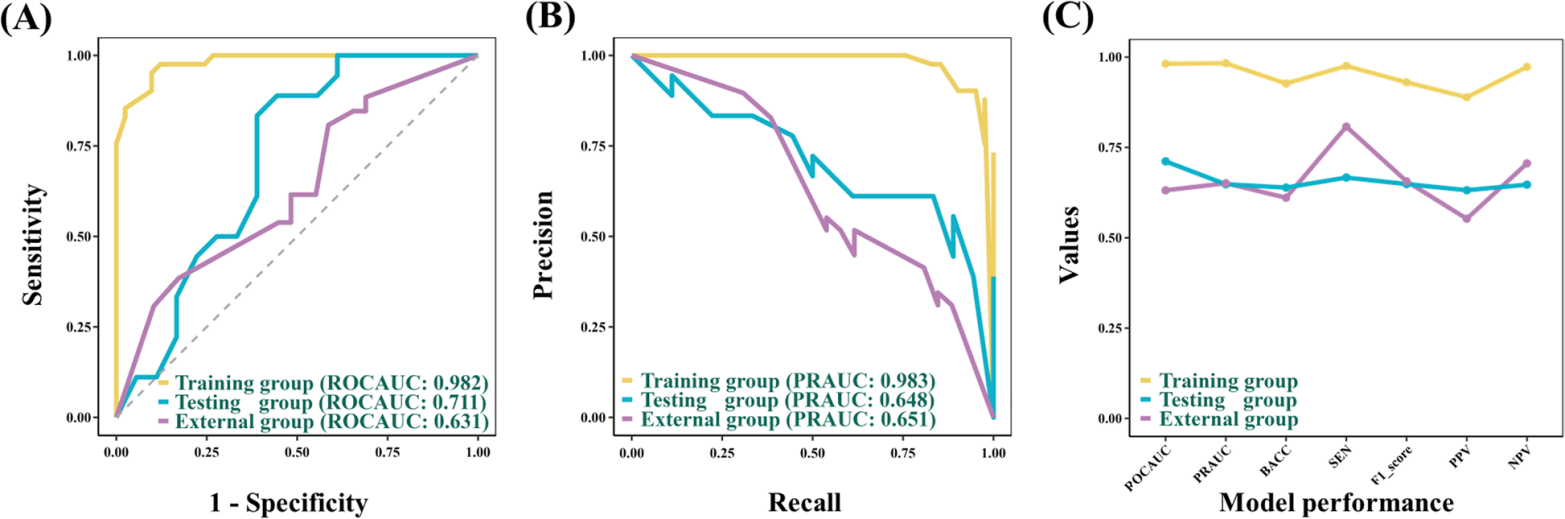
Receiver operating characteristic and precision-recall curves of the random forest model in the training, testing, and external groups (**A**, **B**). The line charts (**C**) of the random forest model performance metrics: area under the receiver operating characteristic (ROCAUC) curve, area under the precision-recall curve (PRAUC), balance accuracy(BACC), sensitivity (SEN), F1 score, positive predictive value (PPV), and negative predictive value (NPV)

The RF model performance metrics (ROCAUC, PRAUC, BACC, sensitivity, F1 score, PPV, and NPV) of the training and testing groups were 0.982, 0.983, 0.927, 0.976, 0.930, 0.889, and 0.973; and 0.711, 0.648, 0.639, 0.667, 0.649, 0.632, and 0.647, respectively (Table 2 and Fig. 4A, B). Moreover, these of the external group were 0.631, 0.651, 0.611, 0.808, 0.656, 0.553, and 0.706, respectively (Table 2 and Fig. 4C).

**Table 2:** The metrics of random forest prediction models

	ROCAUC	PRAUC	BACC	SEN	F1 score	PPV	NPV
Training group	0. 982	0. 983	0. 927	0. 976	0. 930	0. 889	0. 973
Testing group	0. 711	0. 648	0. 639	0. 667	0. 649	0. 632	0. 647
External group	0. 631	0. 651	0. 611	0. 808	0. 656	0. 553	0. 706

*BACC* balance accuracy, *NPV* negative predictive value, *PPV* positive predictive value, *PRAUC* area under the precision-recall curve, *ROCAUC* area under the receiver operating characteristic, *SEN* sensitivity

Moreover, in the testing group, the KNN and Lightgbm models generally outperform the RF model in terms of multiple metrics. In the external validation group, the DT model achieves a higher PRAUC than the RF model. However, the RF model overall demonstrates the best performance among all external models. The detailed model metrics of all 9 models across the testing and external validation datasets were summarized in the Supplementary Materials (Figure S1 and Table S1).

### Subgroup analysis and correlation analysis results

**Fig. 5:**
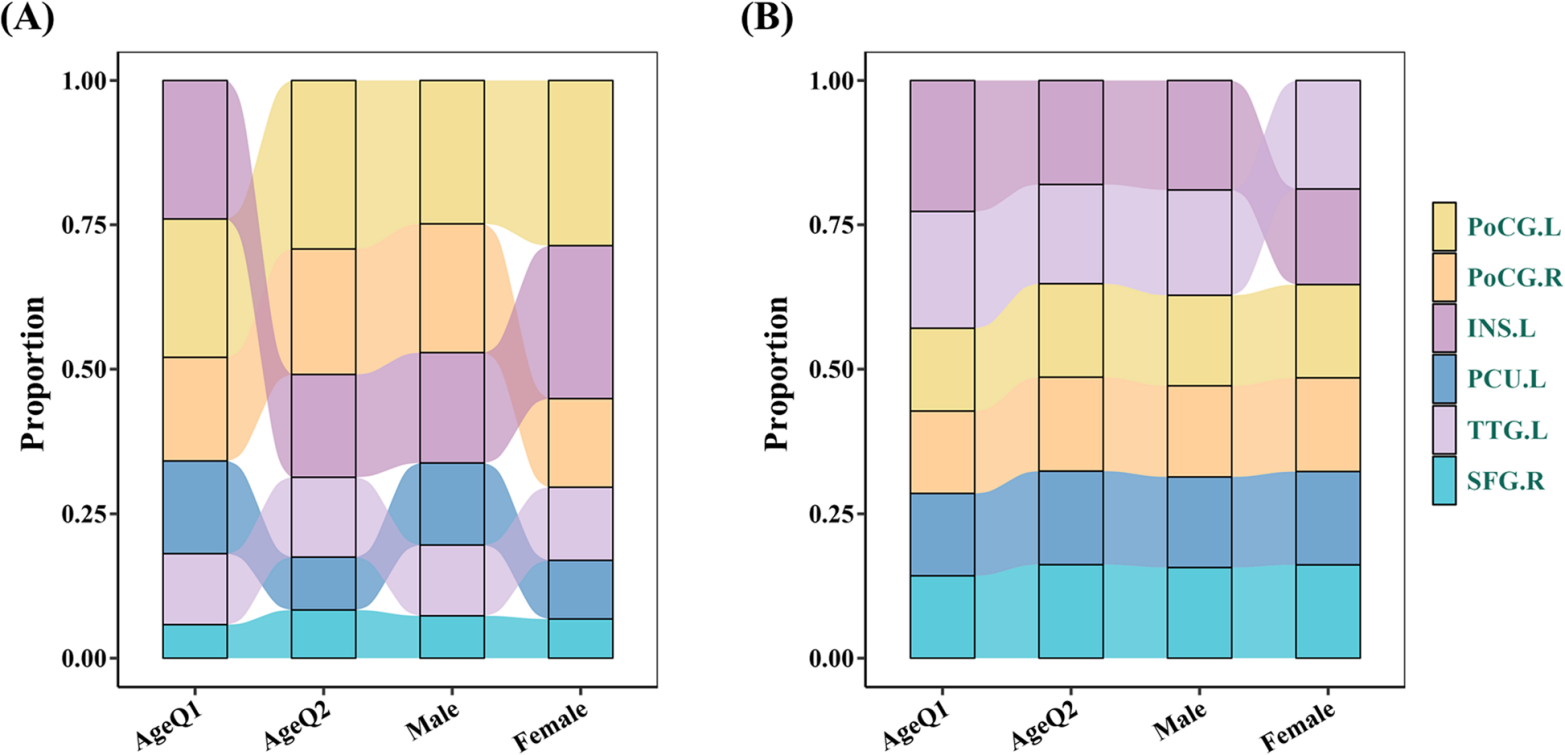
Different contribution proportions of selected features across age and sex subgroups in migraine dataset (**A**) and external dataset (**B**). INS, insula; PCU, precuneus; PoCG, postcentral gyrus; SFG, superior frontal gyrus; TTG, transverse temporal gyrus; L, left; R, right

Subgroup analysis reveals no significant interactions among all subgroups, and the detailed results are summarized in Table 3. Moreover, subgroup analysis results indicate that in patients with episodic migraine, the GMV of the PoCG predominates in the model, followed by the functions of INS and TTG. Conversely, in patients with chronic osteoarthritis, the functions of INS and TTG become the primary contributors, followed by the GMV of the PoCG. The importance of PCU and SFG remains relatively stable in all subgroups. The detailed varied contribution levels of these features in age and sex subgroups of our migraine and external validation datasets are described in Fig. 5A, B.

**Table 3:** Subgroup analysis results

Variables	Count	Percent (%)	OR (95% CI)	*p* value	*p* for interaction
**Overall**	118	100.00	0.19 (0.09 ~ 0.41)	< 0.001	
**Age**					0.087
≤ 33 years	60	50.80	0.09 (0.03 ~ 0.29)	< 0.001	
> 33 years	58	49.20	0.38 (0.13 ~ 1.08)	0.073	
**Sex**					0.231
Male	20	16.90	0.05 (0.02 ~ 0.41)	0.016	
Female	98	83.10	0.23 (0.10 ~ 0.54)	0.001	
**Education**					0.903
≤ 12 years	60	50.80	0.18 (0.06 ~ 0.53)	0.003	
> 12 years	58	49.20	0.20 (0.06 ~ 0.59)	0.005	
**Migraine duration**					0.159
≤ 8 years	64	54.20	0.11 (0.03 ~ 0.33)	< 0.001	
> 8 years	54	45.80	0.35 (0.11 ~ 1.02)	0.060	
**VAS**					0.839
≤ 6 score	64	54.20	0.17 (0.06 ~ 0.50)	0.002	
> 6 score	54	45.80	0.21 (0.06 ~ 0.64)	0.008	
**Frequency**					0.217
≤ 3 days/month	59	50.00	0.11 (0.03 ~ 0.34)	< 0.001	
> 3 days/month	59	50.00	0.30 (0.10 ~ 0.88)	0.032	
**Attack duration**					0.126
≤ 12 h	63	53.40	0.33 (0.11 ~ 0.90)	0.033	
> 12 h	55	46.60	0.09 (0.02 ~ 0.30)	< 0.001	
**MIDAS**					0.416
≤ 20 score	59	50.00	0.13 (0.04 ~ 0.41)	0.001	
> 20 score	59	50.00	0.26 (0.08 ~ 0.75)	0.015	
**HIT-6**					0.871
≤ 62 score	61	51.70	0.20 (0.06 ~ 0.58)	0.004	
> 62 score	57	48.30	0.18 (0.05 ~ 0.53)	0.003	
**GAD-7**					0.496
≤ 5 score	60	50.80	0.25 (0.08 ~ 0.71)	0.011	
> 5 score	58	49.20	0.14 (0.04 ~ 0.44)	0.001	
**PHQ-9**					0.228
≤ 7 score	69	58.50	0.28 (0.10 ~ 0.75)	0.013	
> 7 score	49	41.50	0.10 (0.03 ~ 0.36)	0.001	
**PSQI**					0.847
≤ 7 score	37	31.40	0.17 (0.03 ~ 0.66)	0.015	
> 7 score	81	68.60	0.20 (0.07 ~ 0.49)	0.001	

*GAD-7* Generalized Anxiety Disorder 7-Item, *HIT-6* Headache Impact Test 6-Item, *MIDAS* Migraine Disability Assessment Scale, *PHQ-9* Patient Health Questionnaire 9-Item, *PSQI* Pittsburgh Sleep Quality Index, *VAS* Visual Analogue Scale

**Fig. 6:**
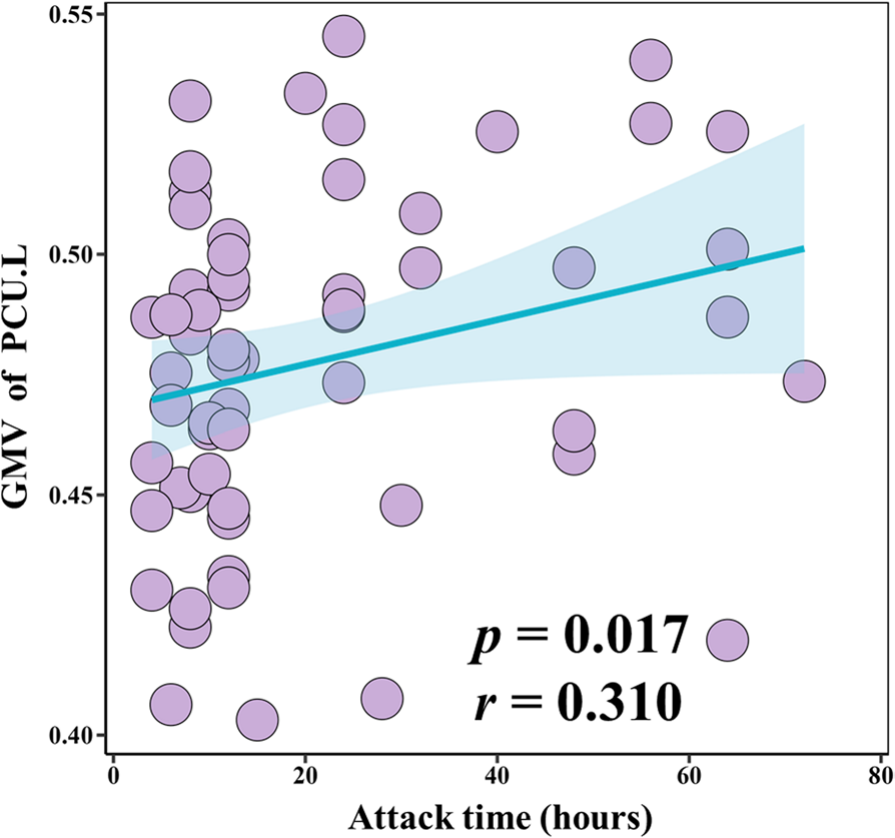
Positive correlation between the GMV of the left PCU and attack time (*r* = 0.310, *p* = 0.017). GMV, gray matter volume; PCU, precuneus; L, left

In the non-response group, the GMV of the PCU.L was positively correlated with attack time (*r* = 0.310, *p* = 0.017) (Fig. 6). No other significant correlations were found.

## Discussion

In this study, we developed a RF model for the analgesic effect of episodic migraine treatment based on the whole-brain functional and structural signatures. Although some other models exhibited superior performance than the RF model in terms of certain prediction metrics in the testing and external groups, it is essential to consider that during the model construction and selection phase, the prediction metrics for both the testing and external groups were unknown. This model also predicted an independent dataset in patients with chronic knee osteoarthritis pain [39], and showed comparable performance. The selection of this specific external validation dataset was guided by several critical factors. First, the experimental design closely resembled our study, involving multimodal magnetic resonance imaging scanning prior to a 3-month treatment period and the primary objective to predict post-treatment analgesic effects based on baseline neuroimaging data. This similarity ensures that the external validation is meaningful. Second, the patient population in the external validation cohort suffers from chronic knee osteoarthritis, a condition that, like migraine, falls under the category of chronic inflammatory diseases and shares certain overlapping pathological mechanism [40]. The existence of this overlap was critical to the selection of this dataset to evaluate the model performance beyond the specific context of migraine. Lastly, the inclusion of both responders and non-responders in the external validation dataset is invaluable. It allows us to evaluate the model performance across different treatment strategies in different pain conditions. Additionally, these datasets were heterogeneous in terms of pain conditions, scanning parameters, and participant characteristics. The heterogeneity of the samples might reduce predictive accuracy during external validation, but it helps develop a model with better robustness and generalizability. Comparisons between models for treatment efficacy in different pain conditions provided evidence that different pain conditions share common brain representations in response to pain treatment. Therefore, this study provides promising neuroimaging biomarkers for predicting the analgesic effect of migraine management with potential for clinical translation.

The observed neuroimaging features were involved in multiple cerebral networks which constitute a complex TVS [13]. These networks mainly include the default mode network (DMN), central executive network (CEN), auditory network, sensorimotor network (SMN), and salience network (SN). The SFG is part of the prefrontal cortex (PFC), and together with the parietal cortex constitutes the CEN typically associated with the cognitive and executive functions [41, 42]. The PCU is a core region of the posterior DMN (pDMN), which is highly active at rest and inhibits activity mainly involved in internally-directed cognition, such as emotional processing and self-referential activity [43, 44]. The INS is a hub in the SN, which is mainly responsible for regulating attention shifting between the exogenous and endogenous states [45–47]. The PoCG belongs to the primary sensory cortex (S1) and combines the premotor cortex, primary motor cortex, and posterior parietal cortex to receive and integrate motor and sensory information for planning and coordinating complex movements [48, 49]. The TTG also known as Heschl’s gyrus is the typical location of the primary auditory cortex and is the first cortical structure to process incoming auditory information [50]. However, previous research indicated that the TTG is a part of the SMN [51]. In this study, our results suggest that the functional and structural abnormalities of TVS may play a crucial role in the modulation of pain-related signals and in our model of predicting analgesic effect, although this hypothesis needs to be tested more thoroughly in the future.

The key finding of the present study is that aberrant multimodal neuroimaging features in the TVS are pivotal factors in the neuropathological and analgesic mechanisms of migraine. The TVS is primarily composed of an ascending pain facilitatory pathway and a descending pain modulatory pathway [13]. Similar to our results, a previous study has shown that compared with HCs, individuals with migraine exhibited higher signal variation in the ascending trigeminal spinal-thalamo-cortical pathway, mainly including the spinal trigeminal nucleus, thalamus, S1, and posterior INS. Conversely, they exhibited lower signal variation in the descending pain modulatory pathway, mainly located in the dorsolateral PFC and parietal cortex [52]. Moreover, some resting-state functional MRI studies, focusing on pain conditions such as trigeminal neuralgia [53], postherpetic neuralgia [54], and chronic back pain [55], have shown reduced neural rhythms in the PFC and its associated projection pathways to the mesolimbic system, suggesting that PFC inhibition may represent a maladaptive cortical process derived from the mechanisms of persistent pain [56]. Therefore, the above findings indicated that patients with migraine showed the ability of excessive upward transmission and insufficient downward regulation of nociceptive signals. This regulatory imbalance of pain signals in the ascending descending pathways is considered the core neural mechanism underlying the occurrence, development, and analgesia of migraine.

The research has also shown that the results of the SHAP plot and subgroup analysis are similar, both indicating that the PoCG and INS play a pivotal role in the prediction models. In recent studies using resting-state functional and structural MRI, various brain networks with functional and structural changes have been reported in patients with migraine, and show notable correlations with the clinical features [57, 58]. These brain networks are consistent with the brain regions observed in this study. Moreover, there is growing evidence that neurological functional change and tissue morphology change do not synchronize, and changes in brain function are likely to be more sensitive than those in structural pathways [59–61]. These results implied a causal dissociation between functional impairment and structural damage. In the subgroup analysis, we observed that the younger migraine cohort showed the most significant contribution in terms of the INS function, whereas the older migraine cohort exhibited a heightened contribution degree concerning GMV within the S1, followed by the INS function. Notably, the external cohort, which consisted of individuals of advanced age in comparison to the migraine cohort, exhibited more importance of functional alterations in all subgroups. Especially, the younger subgroup of the external cohort had a relatively higher contribution proportion of function to treatment effects, similar to the episodic migraine cohort. This phenomenon may be attributed to age-related changes in brain structural integrity, which subsequently exert an influence on function.

Despite the general trend of age-related changes in cerebral cortex, such as a decrease in volume or thickness, particularly with a rate of gray matter loss in the INS that is twice the average rate across the entire brain [62], it has been observed that the structure of the INS does not significantly change with age in patients with migraine [63]. Compared to healthy female controls, female patients with migraine have thicker INS and PCU, while no such changes were found in male patients with migraine [64]. These suggested that resistance of cortical atrophy in INS is an inherent feature of migraine and functional alterations in INS render it more sensitive to the pathological mechanisms in migraine, especially in female population. Moreover, a positive correlation between larger GMV (including S1 and INS) and pain tolerance suggested that brain structure may have a neuroplastic compensatory function for recurrent stress in a general population [65]. Therefore, for the heterogeneity of the structural and functional contributions to the therapeutic response between migraine and osteoarthritis groups in the subgroup results, possible explanations include the decompensation of aging brain structure in response to long-term chronic stimulation by pain signals and physiological atrophy, leading to a more important role of function regulation in analgesic effects in older external group. Further, we speculated that individual variations in pain perception and therapeutic outcomes may be attributed to the distinct processing of pain signals and neuromodulatory mechanisms in the brain, which are characteristic of various pain conditions.

In addition, a study on the neurological mechanism of NSAIDs antinociception using arterial spin labeling found that pain condition significantly increased cerebral blood flow (CBF) in various brain regions such as the brainstem, midbrain, thalamus, INS, S1, and PFC. However, after administering ibuprofen, CBF in the INS, S1, and PFC decreased significantly [20]. An animal model has also shown that NSAIDs-evoked antinociception by injecting into the INS can significantly reduce the reaction to exogenous pain stimuli via descending endogenous opioid and cannabinoid system [66]. Moreover, the INS can predict the response to transcutaneous vagus nerve stimulation and showed a negative correlation between the decreased fractional amplitude of low frequency fluctuations (fALFF) and the relief of migraine symptoms [67]. The fALFF, which is the ratio of ALFF values at specific frequency bands (0.01 Hz to 0.08 Hz) to the overall ALFF value, can effectively reduce noise interference and improve the sensitivity and specificity of spontaneous brain activity detection. The perAF adopted is similar, reflecting fluctuation in neural activity [29]. Lower values indicate more stable neural function. We also found a negative contribution of the perAF of INS to predict NSAIDs efficacy in migraine. These results suggest that the stable functional activity observed in the INS serves as a preparation for and defense against the impact of pain signals. This stable functional activity may represent a self-protective response of the brain to painful stimuli. Such a response likely involves a range of neurobiological and physiological mechanisms aimed at maintaining internal homeostasis and balance within the body. These findings further underscore the complexity of pain processing in the brain and the significant role played by the INS in this process.

Previous brain neuroimaging studies focusing on pain have identified a pain matrix [68, 69], which consists of the S1, INS, secondary somatosensory cortex, PFC, and limbic system, representing the perceived intensity and unpleasantness elicited by nociceptive stimuli [70]. The S1, secondary sensory cortex, and posterior INS of the pain matrix were associated with the sensory component of nociception, whereas the anterior INS was associated with the affective component of nociception [13, 71]. Moreover, the S1and PFC are the initial regions of the descending pain regulation pathway, which may directly or indirectly act on the trigeminal nerve complex via the trigemino-thalamo-cortical nociceptive pathway to exhibit the regulatory function of the input nociceptive signals [13, 72]. It has been pointed out that abnormal functional connectivity patterns between the S1 and PFC may imply dysfunction in the encoding of pain sensory and affective dimensions [56, 73], which may lead to regulatory deficits in the descending pathway and cause prolonged pain states. Furthermore, recent neuroimaging studies on whole-brain functional connectivity analysis showed that the abnormal functional connectivity patterns of the pain matrix played an important role in chronic migraine progression [68, 74]. Moreover, an arterial spin labeling study showed that NSAIDs achieved analgesia by inhibiting neural activity in the brain regions of the descending pain regulatory pathway [20]. In addition, patients with migraine experience a loss of volume in the left somatosensory cortex [57], suggesting larger volume of the left S1 positively contributes to predicting therapeutic outcomes, indicating a compensatory mechanism for responding to pain signals [65]. Based on our findings, we inferred that the altered functional and structural characterizations of brain regions in the descending pain regulatory pathway were involved in the neural mechanism of migraine progression and pharmacological treatment.

The present study is of great importance because it enhanced our understanding by revealing that the multimodal neuroimaging features of the TVS possess the potential to serve as neural biomarkers for migraine treatment. Such insights deepen the current understanding of migraine-related neuropathological mechanisms and medication-related analgesic mechanisms. We believe that this study may contribute to the field forward because these findings may assist in providing precise clinical interventions to shorten the disease course and prevent further deterioration. Furthermore, with the increasing use of NSAIDs worldwide [7], the ability to predict the outcome of NSAIDs treatment would facilitate the development of individualized treatment plans and promote their wider application. In addition, previous research has suggested that the functional and structural alterations present in patients with migraine can serve as predictive indicators for the efficacy of migraine treatments [75–77]. Although the neuroimaging characteristics of these predictive brain regions exhibit some degree of consistency with our results, there remains a lack of a cohesive neural mechanism to effectively elucidate the combined influence of functional and structural factors on treatment efficacy. These investigations indicate that the impact of pain on the brain entails intricate interactions between functional and structural components, resulting in enduring alterations in pain perception, emotional reactivity, and cognitive processing, particularly pronounced in individuals experiencing chronic pain. As such, effective pain management strategies should acknowledge these complex cerebral changes and seek integrated approaches to mitigate pain and enhance overall quality of life.

This study has certain limitations that need to be addressed to enhance its overall validity and reliability. First, it is a single-center study with a limited amount of data. External validation was conducted with other pain disorders rather than migraine for predicting the analgesic effects. Conducting multicenter studies, including external NSAIDs-treated migraine cohorts, increasing sample sizes, and improving the stability, credibility, and generalizability of existing data are imperative in future research. Second, although the study predicted the outcome of NSAIDs based on baseline data, it could not reflect the causal relationship between changes in neuroimaging characteristics and the effects of drugs. Therefore, further longitudinal studies are needed to overcome this limitation. Third, self-reported drug consumption information and the lack of compliance may affect the effectiveness of the study. Due to the low compliance with oral analgesics, the establishment of stringent and standardized treatment protocols is challenging. This is particularly true for patients with migraine, who frequently modify their drug dosage in response to the perceived severity of pain during headache episodes. Addressing these issues is essential for the advancement of migraine treatment research. Lastly, chronic pain syndromes are often accompanied by comorbid psychological and psychiatric conditions. Despite our efforts to balance anxiety and depression scores between the two migraine groups, the quantification of participants' psychological states within a rigorous quantitative framework continues to pose a substantial challenge [78]. Computational psychophysiology, an emerging methodology, offers a novel pathway to decipher the intricate associations linking psychological and physiological parameters [79]. This approach is particularly well-suited to the advancement of personalized and precision medicine. The incorporation of computational psychophysiology to develop predictive models for treatment outcomes represents a promising frontier for future research, deserving of in-depth investigation and refinement.

## Conclusions

In conclusion, this study explored potential neuroimaging biomarkers of nociceptive modulation and showed that the multimodal features of the TVS had promising prospects for predicting the efficacy of NSAIDs in individual episodic migraine management. This will help orient pharmacological strategies and optimize the distribution of medical resources, thereby achieving the ultimate goal of enhancing migraine treatment outcomes.

### Supplementary Information

Supplementary Material 1.

## Data Availability

The data used and analyzed in this study are available from the corresponding author upon reasonable request.

## Abbreviations

BACC: Balance accuracy
CBF: Cerebral blood flow
CEN: Central executive network
DMN: Default mode network
DT: Decision tree
ENet: Elastic network
fALFF: Fractional amplitude of low frequency fluctuations
GAD-7: Generalized Anxiety Disorder 7-Item
GMV: Gray matter volume
HIT-6: Headache Impact Test 6-Item
ICHD: International Classification of Headache Disorders
INS: Insula
KNN: K-nearest neighbor
LASSO: Least absolute shrinkage and selection operator
Lightgbm: Light gradient boosting machine
LR: Logistic regression
MIDAS: Migraine Disability Assessment Scale
ML: Machine learning
MLP: Multilayer perceptron
MNI: Montreal Neurological Institute
NPV: Negative predictive value
NSAIDs: Non-steroidal anti-inflammatory drugs
PCU: Precuneus; PerAF, percent amplitude of fluctuation
PFC: Prefrontal cortex
PHQ-9: Patient Health Questionnaire 9-Item
PoCG: Postcentral gyrus
PPV: Positive predictive value
PRAUC: Area under the precision-recall curve
PSM: Propensity score matching
PSQI: Pittsburgh Sleep Quality Index
RF: Random forest
RMS: Root mean square
ROCAUC: Area under the receiver operating characteristic
S1: Primary sensory cortex
SFG: Superior frontal gyrus
SMN: Sensorimotor network
SN: Salience network
SVM-RFE: Support vector machine recursive feature elimination
TTG: Transverse temporal gyrus
TVS: Trigeminovascular system
VAS: Visual Analogue Scale
Xgboost: Extreme gradient boosting
