# Supplementary material for: Exploring potential neuroimaging biomarkers for the response to non-steroidal anti-inflammatory drugs in episodic migraine
Source: J Headache Pain. 2024 Jun 21;25(1):104. doi: 10.1186/s10194-024-01812-4 (PMC11191194; doi:10.1186/s10194-024-01812-4)
Supplement: Supplementary file 1 — Supplementary Material 1. [file 10194_2024_1812_MOESM1_ESM.docx]

**Inclusion and exclusion criteria**

To control the potential pharmacological and physiological effects, the key inclusion criteria were: (1) patients were drug-free for at least 1 month before being enrolled, (2) patients in the interictal phase were headache-free for at least 3 days before and after scanning, ascertained by a structured telephonic interview, and (3) patients were not taking any medicines (except for non-steroidal anti-inflammatory drugs) during follow-up period. The exclusion criteria were (1) comorbidity with other forms of headache and neuropsychological or neurological diseases, (2) previous brain injury or psychoactive medication use, (3) a history of alcohol or drug abuse, (4) pregnant or lactating women, and (5) any contraindications to MRI scanning.

**MRI scanning parameters**

Data from multimodal MRI were acquired using a 3.0-Tesla MRI scanner (Philips, Ingenia) with an eight-channel head coil. For this analysis, the functional images were acquired axially using a gradient echo-planar imaging sequence as follows: repetition time (TR) = 2,000 ms; echo time (TE) = 30 ms; slices = 36; thickness = 4 mm; gap = 0 mm; field of view (FOV) = 240 mm × 240 mm; acquisition matrix = 64 × 64; and flip angle (FA) = 90º. The functional sequence took 8 min and 8 s. Moreover, structural images were obtained using a three-dimensional turbo fast echo T1WI sequence with the following parameters: TR/TE = 8.1/3.7 ms; slices = 170; thickness = 1 mm; gap = 0 mm; FA = 8º; acquisition matrix = 256 × 256; FOV = 256 mm × 256 mm. The structural sequence took 5 min and 29 s. During the scanning, the scanner noise and head motion were reduced using earplugs and foam padding, and the participants were instructed to reflex and lie with their eyes closed but not fall asleep.


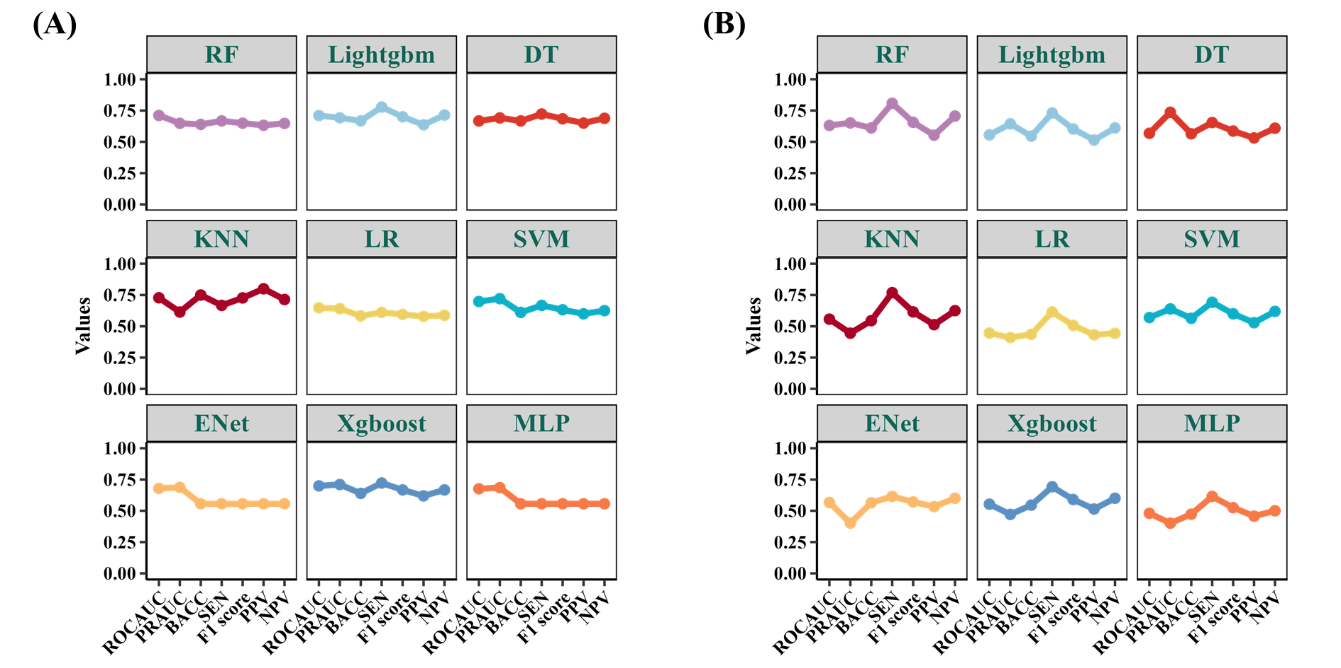


**Figure S1.** The line charts representing the metrics of all 9 models in testing group (**A**) and external validation group (**B**). BACC, balance accuracy; DT, decision tree; ENet, elastic network; KNN, k-nearest neighbor; Lightgbm, light gradient boosting machine; LR, logistic regression; MLP, multilayer perceptron; NPV, negative predictive value; PPV, positive predictive value; RF, random forest; ROCAUC, area under the receiver operating characteristic; SVM, support vector machine; Xgboost, extreme gradient boosting.

| **Table S1.** The metrics of all 9 models across the testing and external groups. | | | | | | | | |
| --- | --- | --- | --- | --- | --- | --- | --- | --- |
| **Groups** | **Models** | **ROCAUC** | **PRAUC** | **BACC** | **SEN** | **F1 score** | **PPV** | **NPV** |
| **Testing** | LR | 0.648 | 0.641 | 0.583 | 0.611 | 0.595 | 0.579 | 0.588 |
|  | SVM | 0.698 | **0.721** | 0.611 | 0.667 | 0.632 | 0.600 | 0.625 |
|  | RF | 0.711 | 0.648 | 0.639 | 0.667 | 0.649 | 0.632 | 0.647 |
|  | DT | 0.667 | 0.691 | 0.667 | 0.722 | 0.684 | 0.65 | 0.688 |
|  | KNN | **0.728** | 0.614 | **0.750** | 0.667 | **0.727** | **0.800** | **0.714** |
|  | MLP | 0.676 | 0.685 | 0.556 | 0.556 | 0.556 | 0.556 | 0.556 |
|  | ENet | 0.679 | 0.687 | 0.556 | 0.556 | 0.556 | 0.556 | 0.556 |
|  | Lightgbm | 0.710 | 0.692 | 0.667 | **0.778** | 0.700 | 0.636 | **0.714** |
|  | Xgboost | 0.699 | 0.710 | 0.639 | 0.722 | 0.667 | 0.619 | 0.667 |
| **External** | LR | 0.411 | 0.410 | 0.400 | 0.615 | 0.492 | 0.410 | 0.375 |
|  | SVM | 0.570 | 0.639 | 0.564 | 0.692 | 0.600 | 0.529 | 0.619 |
|  | RF | **0.631** | 0.651 | 0.611 | **0.808** | **0.656** | 0.553 | **0.706** |
|  | DT | 0.568 | **0.736** | 0.564 | 0.654 | 0.586 | 0.531 | 0.609 |
|  | KNN | 0.411 | 0.445 | 0.400 | 0.615 | 0.492 | 0.410 | 0.375 |
|  | MLP | 0.480 | 0.401 | 0.473 | 0.615 | 0.525 | 0.457 | 0.500 |
|  | ENet | 0.566 | 0.402 | 0.564 | 0.615 | 0.571 | 0.533 | 0.600 |
|  | Lightgbm | 0.555 | 0.644 | 0.545 | 0.731 | 0.603 | 0.514 | 0.611 |
|  | Xgboost | 0.519 | 0.471 | 0.509 | 0.692 | 0.571 | 0.486 | 0.556 |
| BACC, balance accuracy; DT, decision tree; ENet, elastic network; KNN, k-nearest neighbor; Lightgbm, light gradient boosting machine; LR, logistic regression; MLP, multilayer perceptron; NPV, negative predictive value; PPV, positive predictive value; RF, random forest; ROCAUC, area under the receiver operating characteristic; SVM, support vector machine; Xgboost, extreme gradient boosting. | | | | | | | | |
